# The Week's Work

**Published:** 1910-07-23

**Authors:** 


### The Week's Work.

### AN IMPRACTICABLE NOTION.

A correspondent, who no doubt means excellently, but can have little practical acquaintance with the organisation and business management of a large hospital, has written a letter to the *Daily Mail*, which was published in the issue of June 25, suggesting a method for aiding voluntary hospitals. It has occurred to him, he says, that a large sum of money is expended on secretarial and clerical work which might be voluntarily discharged by an honorary staff in much the same way as the services of the professional staff are given. He goes on to state that there must be many who would be able and willing to undertake the duties. The analogy thus drawn between the clerical and medical staffs of a hospital is, of course, entirely illusory. The members of the visiting medical staff do not give up the whole of their time to the service of the hospital; obviously if they did they must be all men of extensive private means, since their services are gratuitous. But, in point of fact, their services are rendered at certain limited hours, and sometimes only on fixed days of the week. Moreover, they are not amateurs at the work, but professionals of the highest skill. Contrast this with the proposal for utilising well-meaning but untrained assistance on the clerical staff. How can the affairs of a complex business concern such as a hospital possibly be conducted by a staff of amateur clerks, giving each a few hours a week to their duties? As for that, how can such voluntary helpers even guarantee their attendance at regular hours? If of independent means, the routine and the hard work would very soon begin to pall; and if the workers are already earning a living in some other occupation, how can they possibly offer efficient service to the institution?

### HOME VISITATION FROM THE HOSPITALS.

This was the title of an interesting paper read by Miss Mudd, a London hospital worker, before the nineteenth annual National Conference of the Charity Organisation Societies, held at Leeds. Miss Mudd considers that it is imperatively necessary for the charity visitor to study the conditions of family life among those with whom the almoner came into contact. The paper dealt with the general aim and scope of almoners' duties, a subject to which we have already devoted some attention in these columns. Home visitation is comparatively novel, and few provincial hospitals at present possess almoners, but the usefulness of these officials is now generally admitted by hospital workers. The Training Council is doing good work in enlisting recruits and providing them with proper facilities to become thoroughly familiar with the duties, responsibilities, and general work of institutional almoners. The difficulty, which no amount of training can overcome, is to find men and women who are sympathetic and who possess the proper "almoning spirit." It is difficult to define the qualities that a good almoner should possess, but a bad almoner, by frightening off patients and by interfering with the staff and the authorities, sometimes creates more difficulties than she finds already in existence when she commences her work. For the smooth working of the almoning system officials are required who are not only officials but who are real charitable workers. As Miss Mudd points out, it is therefore of the greatest importance that all would-be almoners should have a thorough training and a comparatively long probationary period, while, above all, they must become thoroughly acquainted with the conditions under which the poor exist.

### THE MINORITY MAN—AND WOMAN.

To believe against the belief of the majority is an ineradicable instinct of some minds. Rarely the belief may be justified, as with Galileo, or the pre-Raphaelites, or people who bought railway shares in the forties of last century. But as, unfortunately, error abounds rather than truth, vastly the more common thing is for such isolated faith to be false, and a lamentable hindrance to real progress. Perhaps nowhere can more signal instances of this human trait be found than in medicine. The world, as Pater said, conscious of its ills, is patient of all manner of doctrinaires with plans for a cure. *A fortiori* (since the individual is more keenly concerned for self than the mass is), a sick man will look with attentive eyes on any proposal whatsoever which mentions putting him on his legs again. And ever since man acquired legs to be put upon, the interest and sympathy of his fellows have raised up plenty of active, if not always intelligent, therapeutic ministrants. But of all the countless notions in this line emanating from those outside the regular tradition of medicine, notions which often had immense vogue among the laity and made their authors wealthy, it is a sad reflection for humane people—and for political economists and social reformers—how uniformly the test of time has shown them to have been nonsense. It would be absurd to think all this is going to end in the twentieth century.

### RUNNING DOWN SANATORIUMS.

Individual projects of no intrinsic worth must, however, disappear. If the body of the medical profession hold a right view, then the current drug "cures" of consumption will go the way of their numberless predecessors. There is "no drink or stink," says a plain-spoken Teuton, which has not at one time or another been recommended for this disease. If, on the contrary, the truth lies with Mr. Alabone's friends, who have lately been using the correspondence columns of the *Christian World*, then many sanatorium buildings will be to let before long. For our opinion on the matter we have already given cogent reasons, and to discuss individual cases reported by lay persons is waste of time. One letter from a medical man, we might note, would have been more impressive if the writer had not appended to his not over rare qualifications the style and title "Consultant." The issue may very well be left to the arbitration of time. Meanwhile we would invite the attention of shrewd people to signs already evident of what that arbitration will decree. Nearly every month a paragraph in the newspapers announces the opening or the beginning of some new sanatorium. Queen Alexandra's Institution at Davos has just been given a needed donation of £25,000, and two sanatoriums in Northumberland more than half that sum. Abroad the same thing happens. Dr. Hermann Biggs told the recent Tuberculosis Conference at Edinburgh, that New York was building a municipal sanatorium for a thousand patients. Let us take an illustration which will appeal to readers of the *Christian World*. In its early days the Salvation Army came in for hot abuse—much hotter than has ever been bestowed on sanatoriums. Only a few years ago the writer heard imputations of dishonesty on "General" Booth's part made with impunity on a public stage. The highly effective answer this organisation made was to go on growing rapidly. So perhaps in a less degree with the open-air treatment. It is by no means perfect, but it does far more good to the consumptive than anything else has ever done, and therefore, until knowledge makes another advance, it will supplant everything else.

### WHO BENEFITS?

In our issue of June 25 we called attention to four advertisements which appeared in certain morning papers, each acknowledging a five-pound banknote from an appeal in a weekly newspaper. The paper which has adopted this method of advertising itself asks us, through its manager, to state that it is the *Gentlewoman*. He seems oblivious or indifferent to the fact that the plan pursued may deceive the public into thinking that such advertisements are inserted by the managers of the charities mentioned. If they had been so inserted it is clear, as we pointed out, that the expense of these advertisements in two morning papers only at the published scale rate of these papers was £7 17s. 6d., or nearly 40 per cent. of the £20 advertised. The giving public is aware of the cost of advertisements, and it is not just to any hospital that advertisements of this kind acknowledging small gifts should be inserted at the secret expense of a newspaper or other advertiser. In the present case the *Gentlewoman* clearly gets the chief benefit of such advertisements. If the *Gentlewoman* wishes to advertise itself, and the managers of a public charity choose to lend their name to enable it to do so, every such advertisement should contain a clear statement of the fact. Most hospitals of any standing would, however, decline to lend their name to any advertiser for such a purpose. This is made clear by a letter published in another column, which we have received from a hospital secretary, who no doubt voices the general opinion of hospital managers on the point.

### THE DRUG MARKET.

Business in drugs and chemicals has been slightly better during the week, although it cannot by any means be called good. As had been expected, manufacturers of codeine have reduced their prices by 8d. per ounce; morphine also tends lower. The opium market is still quiet, and buyers are hoping that prices will further recede as the new crop continues to come on the market. Buchu leaves are again dearer, and at present there appears to be no prospect of a decline in value. Menthol is dearer and in good demand. Ergot is tending higher in price. Linseed is dearer, with an advancing tendency. Cocaine is unaltered in price, but an advance is not improbable. Rhubarb is slightly lower in value. The expected advance in the price of potassium bromide and sodium bromide has not yet taken place.

